# Opposing effects of acute and repeated nicotine exposure on boldness in zebrafish

**DOI:** 10.1038/s41598-020-65382-6

**Published:** 2020-05-22

**Authors:** Rachel Dean, Erika Duperreault, Dustin Newton, Jeffrey Krook, Erica Ingraham, Joshua Gallup, Brian C. Franczak, Trevor J. Hamilton

**Affiliations:** 1 Department of Psychology, MacEwan University, Edmonton, AB T5J 4S2 Canada; 2 Department of Mathematics and Statistics, MacEwan University, Edmonton, AB T5J 4S2 Canada; 3 Department of Mathematical and Statistical Sciences, University of Alberta, Edmonton, AB T6G 2G1 Canada; 4 Neuroscience and Mental Health Institute, University of Alberta, Edmonton, AB T6G 2H7 Canada

**Keywords:** Experimental models of disease, Pharmacology

## Abstract

Nicotine is an addictive compound that activates neuronal nicotinic acetylcholine receptors (nAChRs) and causes behavioural effects that vary with dose, schedule of administration, and animal model. In zebrafish (*Danio rerio*), acute doses of nicotine have been consistently found to have anxiolytic properties, whereas, chronic exposure elicits anxiogenic effects. To date, however, studies on repeated nicotine administration and the effects of nicotine withdrawal have not been well explored using this model. In this study, we administered nicotine with three different dosing regimens: 1. Single exposures of a “high” dose (25, 50, 100, or 400 mg/L) for 3 minutes. 2. Single exposures to a “low” dose (2.5, 5, or 20 mg/L) for one hour. 3. Repeated one-hour exposure to a “low” dose (2.5, 5, or 20 mg/L) for 21 days. The novel object approach test was used to examine boldness based on the tendency of the fish to explore a novel object. Acutely, nicotine significantly increased the time spent approaching the object with both three-minute and onehour durations of exposure, indicating increased boldness. Conversely, after repeated nicotine exposure for 21 days, fish spent less time approaching the object suggesting a decrease in boldness. Distance moved was unaffected one hour after repeated nicotine exposure, yet decreased after a two-day withdrawal period. Our work suggests that nicotine can have opposing effects on boldness that vary based on dosage and schedule of exposure.

## Introduction

The global prevalence of tobacco smoking among adults has declined in recent decades, however, the number of daily smokers has increased due to population growth^1^. The harmful effects of repeated tobacco use (e.g. cancer, stroke and heart disease) result in tobacco use being the leading cause of preventable deaths worldwide^2^ at about 7 million per year^3^. When tobacco is consumed, most commonly by smoking, nicotine enters the central nervous system and binds to neuronal nicotinic acetylcholine receptors (nAChRs) that are normally activated by endogenous acetylcholine^4^. Repeated nicotine use results in modifications to dopamine and acetylcholine pathways on reinforcement circuits in the midbrain and cortex^5^ and causes a pleasurable experience via the activation of these circuits which reinforces self-administration^6^.

Contributing to the addictive nature of nicotine is the effect it has on emotion in humans^7,8^. The acute effect of nicotine on anxiety, however, seems to depend on dose and varies across animal models. In rodent models, low doses of nicotine have anxiolytic effects on social interaction, whereas, high doses have an anxiogenic effect^7^. Low doses of nicotine have also been shown to increase the amount of time some strains of mice spend in a mirrored chamber^9^ as well as the time they spend in the white side of a white/black test box^10^, supporting an increase in boldness at low doses. Other studies, however, have failed to produce evidence that nicotine impacts anxiety and/or boldness levels. For example, mice tested in an elevated plus maze did not differ from controls in their exploration of open arms following exposure to nicotine^11^. To date, rodent models have produced variable outcomes^12^, necessitating further investigation in other model species used in pharmacological research.

In recent years, zebrafish (*Danio rerio*) have become an increasingly popular model organism for a variety of fields, including pharmacological studies^13,14^. Their small size and fecundity allow researchers to house and study them in large numbers, their genome has been sequenced and has high homology with humans (~70%^15^), and their ability to readily absorb water soluble chemicals facilitates drug administration^16^. The effects of nicotine have been examined in zebrafish using a variety of developmental, cognitive, and addiction-based paradigms^17^. Nicotine has been shown to improve performance on learning-based tasks as well as enhance memory in zebrafish. Specifically, nicotine improves spatial discrimination learning^18^ as well as performance in a delayed spatial alternation test, where fish are reinforced for selecting the side of the tank opposite to the one chosen in the previous trial^19^. Additionally, nicotine enhances associative learning when given immediately before exposure to an aversive environment (ie. an arena containing conspecific alarm substance^20^). Nicotine has also been observed to increase the duration of object recognition memory^21^. Higher doses impair memory in rodents^22^ and in zebrafish^19^, however acute nicotine exposure decreases anxiety in zebrafish. Specifically, nicotine reduces the amount of time zebrafish spend at the bottom of a novel tank^23^ and reduces shoal cohesion^24^, suggesting a reduction in anxiety and increase in boldness. Furthermore, nicotine lessens the anxiogenic effects of a conspecific alarm substance by decreasing the frequency of erratic movements^20^.

**Figure 1:**
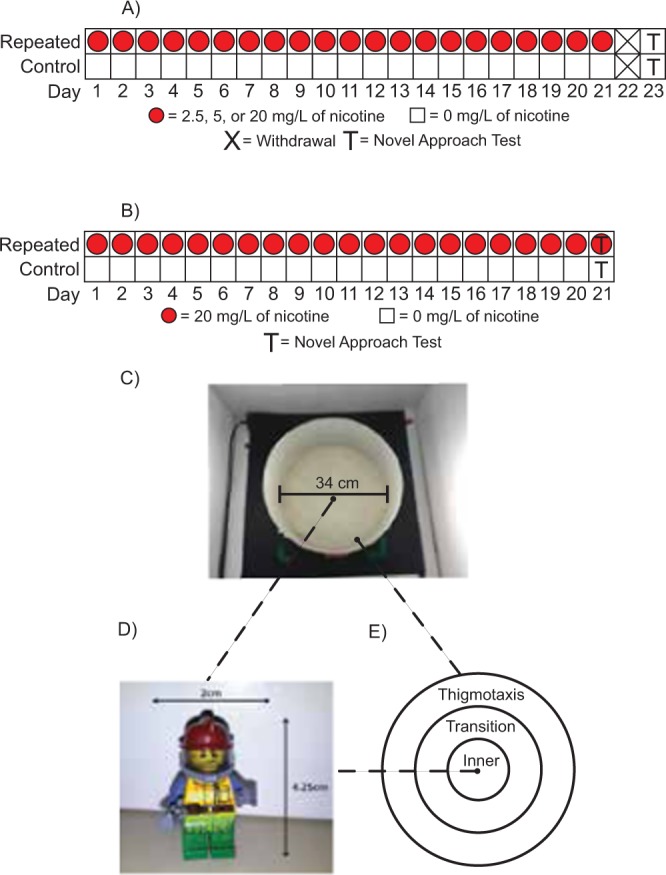
Repeated nicotine administration and novel object approach test apparatus. In RNE experiment 1(**A**), fish in the repeated nicotine groups were placed into dosing tanks containing 2.5, 5, 20 (red circles) or 0 mg/L (white squares) once daily for 21 days. Following the final dose on day 21, fish were left in their housing tanks and were not given nicotine. Fish were then tested (T) on day 23. In RNE experiment 2 (**B**), fish were exposed to 20 (red circles) or 0 mg/L (white squares) of nicotine once daily for 21 days and tested (T) one hour after the final exposure on day 21. For behavioural testing, fish were individually placed into the experimental arena (**C**) which contained the novel object (**D**) in the center of the arena. The arena was divided into three virtual zones (**E**) (Thigmotaxis, Transition, and Inner) for analysis.

To better understand the addictive nature of nicotine, repeated nicotine exposure (RNE) studies have been used in animals to examine behavioural and cellular responses and neuromodulation. Consistent with rodent models^25^, RNE in zebrafish leads to drug-seeking behaviours^26^. A RNE schedule of dosing produces a conditioned place preference for lower concentrations, and a conditioned place aversion for higher concentrations. Following a four-week dosing schedule, the preference for the side of the tank associated with nicotine administration is maintained after three weeks of abstinence as well as its pairing with an aversive stimulus^26^. Furthermore, only three days of RNE is needed to produce a conditioned place preference for an initially aversive environment^27^. On the contrary, zebrafish administered nicotine constantly for prolonged time (chronic) have an anxiogenic response to nicotine. Chronic exposure to nicotine (1–2 mg/L) for a period of four days increases shoal cohesion and the time fish spend at the bottom of a novel tank diving test, both consistent with an anxiogenic effect^28^. As humans do not recreationally consume nicotine chronically, and instead consumption takes place over brief sessions, investigating the behavioural changes from nicotine using a RNE protocol involving daily dosing^29,30^ is more conducive to understanding the effects of nicotine withdrawal in humans. Both humans and rodents exhibit withdrawal symptoms following nicotine cessation^10,31,32^, however, nicotine-induced withdrawal in zebrafish has yet to be explored. In this study, zebrafish were acutely exposed to nicotine for either three minutes or one hour to investigate the dose-response relationship between acute nicotine exposure and boldness. In addition, we examined whether 21 days of RNE would result in altered boldness and locomotion and tested fish following their last nicotine exposure as well as after two days of withdrawal (Fig. 1).

## Results

### Three-minute acute nicotine exposure

**Figure 2:**
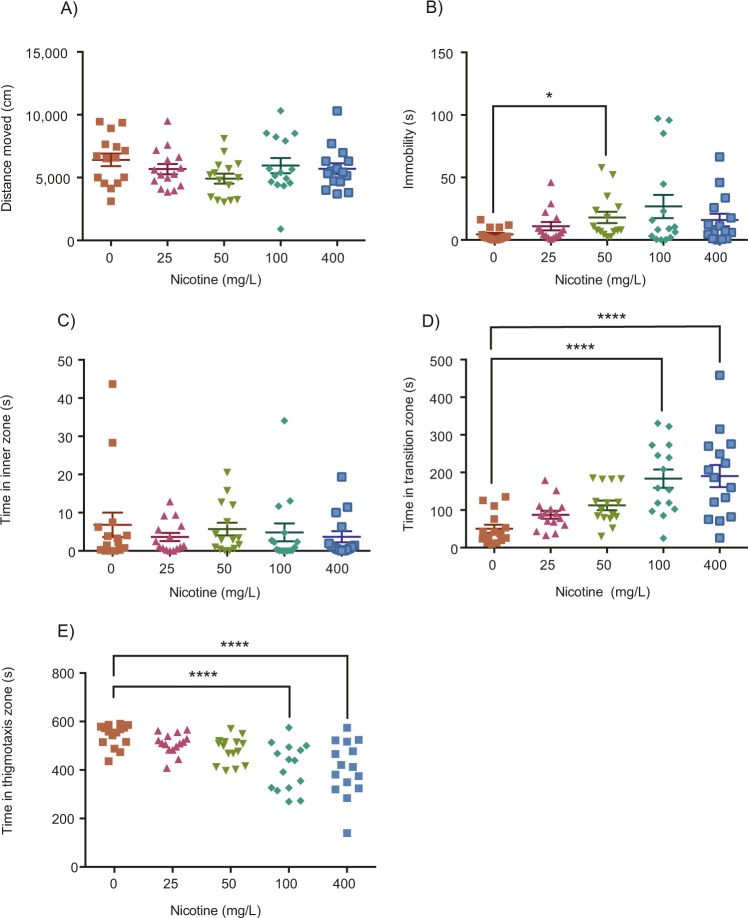
Effect of three minutes of acute nicotine exposure on locomotion and anxiety. Acute exposure to 25, 50, 100, or 400 mg/L did not have a significant effect on distance moved (**A**). Administration of 50 mg/L of nicotine significantly increased time spent immobile (**B**). No effect was found for the time spent in the inner zone (**C**), however, acute exposure to 100 and 400 mg/L of nicotine significantly increased time spent in the transition zone (**D**). Time in the thigmotaxis zone was decreased by 100 and 400 mg/L of nicotine (**E**). ****P < 0.0001; *P < 0.05.

Following an acute three-minute exposure to 0, 25, 50, 100, or 400 mg/L of nicotine, fish were immediately placed into the arena for behavioural testing. No significant effects were found between the 25, 50, 100 or 400 mg/L dosages on total distance moved (Fig. 2A; Kruskal-Wallis; H(4) = 4.380, *p* = 0.3570, η^2^ = 0.005). A significant difference was found between the dosage levels for immobility (Fig. 2B; Kruskal-Wallis; H(4) = 9.660*, p* = 0.0466, η^2^ = 0.081). Post-hoc testing revealed a significant increase in immobility for the 50 mg/L group compared to the control group (Fig. 2B; Dunn’s*; p* = 0.0137, n = 15). No significant effects were found between the dosages for the time spent in the inner zone (Fig. 2C; Kruskal-Wallis; H(4) = 2.054*, p* = 0.7258, η^2^ ≈ 0), however, a significant difference was found between control and nicotine groups in the time spent in the transition zone (Fig. 2D; One-way ANOVA; F(4, 70) = 10.04*, p* < 0.0001, η^2^ = 0.365). Acute exposure to 100 (Fig. 2D; Dunnett’s; *p* < 0.0001, n = 15) and 400 mg/L (Fig. 2D; Dunnett’s; *p* < 0.0001, n = 15) significantly increased the time spent in the transition zone compared to controls. Significant differences were also found between doses for the time spent in the thigmotaxis zone for 100 and 400 mg/L of nicotine (Fig. 2E; One-way ANOVA; F(4, 70) = 9.34*, p* < 0.0001, η^2^ = 0.311).

### 60-minute acute nicotine exposure

**Figure 3:**
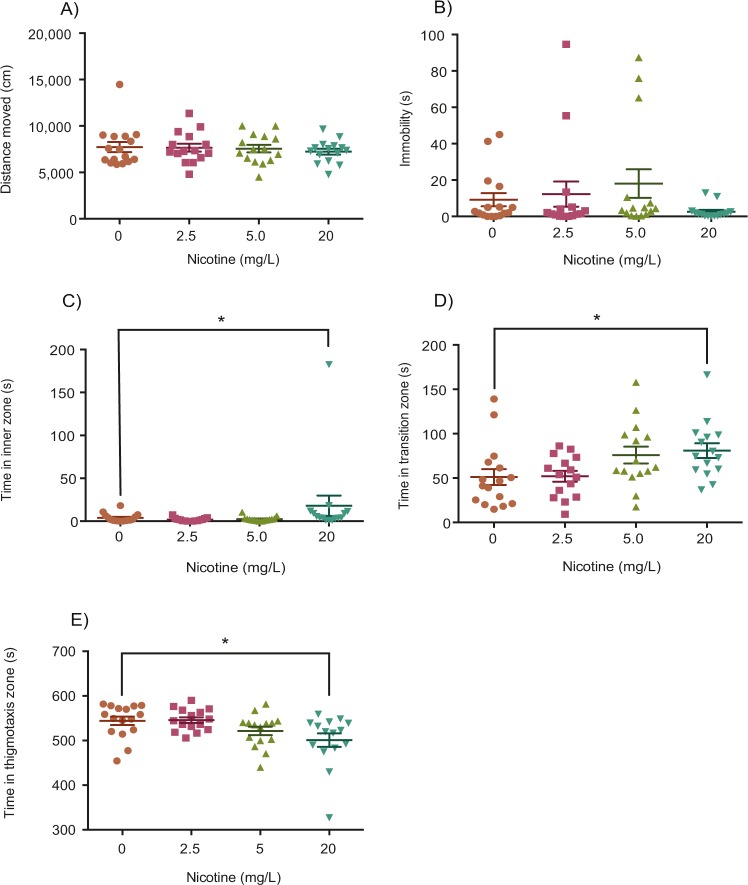
Effect of 60-minutes of acute nicotine exposure on locomotion and anxiety. An acute exposure to 2.5, 5.0, or 20 mg/L of nicotine did not have a significant effect on distance moved (**A**), or immobility (**B**). 20 mg/L of nicotine significantly increased time spent in inner (**C**) and transition zones (**D**) and decreased time spent in the thigmotaxis (**E**) zone. *P < 0.05.

In a separate experiment, zebrafish were exposed to 0, 2.5, 5, or 20 mg/L of nicotine for 60 minutes, then were placed into the arena for behavioural testing. No significant differences were found between nicotine (2.5, 5, or 20 mg/L) and control groups in total distance moved or immobility (Fig. 3A; Kruskal-Wallis; H(3) = 0.3890*, p* = 0.9425, η^2^ ≈ 0; Fig. 3B; Kruskal-Wallis; H(3) = 4.309*, p* = 0.2299, η^2^ = 0.024). These groups were found to differ significantly, however, in the time spent in the inner, transition, and thigmotaxis zones (Fig. 3C; Kruskal-Wallis; H(3) = 11.07*, p* = 0.0113, η^2^ = 0.149; Fig. 3D; Kruskal-Wallis; H(3) = 10.81*, p* = 0.0128, η^2^ = 0.137; Fig. 3E; Kruskal-Wallis; H(3) = 10.72*, p* = 0.0134, η^2^ = 0.151). Post-hoc testing revealed a significant increase in the time spent in the inner (Fig. 3C; Dunn’s*; p* = 0.0474, n = 15) and transition (Fig. 3D; Dunn’s*; p* = 0.0152, n = 15) zones, and a decrease in time spent in the thigmotaxis zone (Fig. 3E, Dunn’s*; p* = 0.0154, n = 15) with the 20 mg/L dose.

### Repeated nicotine exposure – Experiment 1

**Figure 4:**
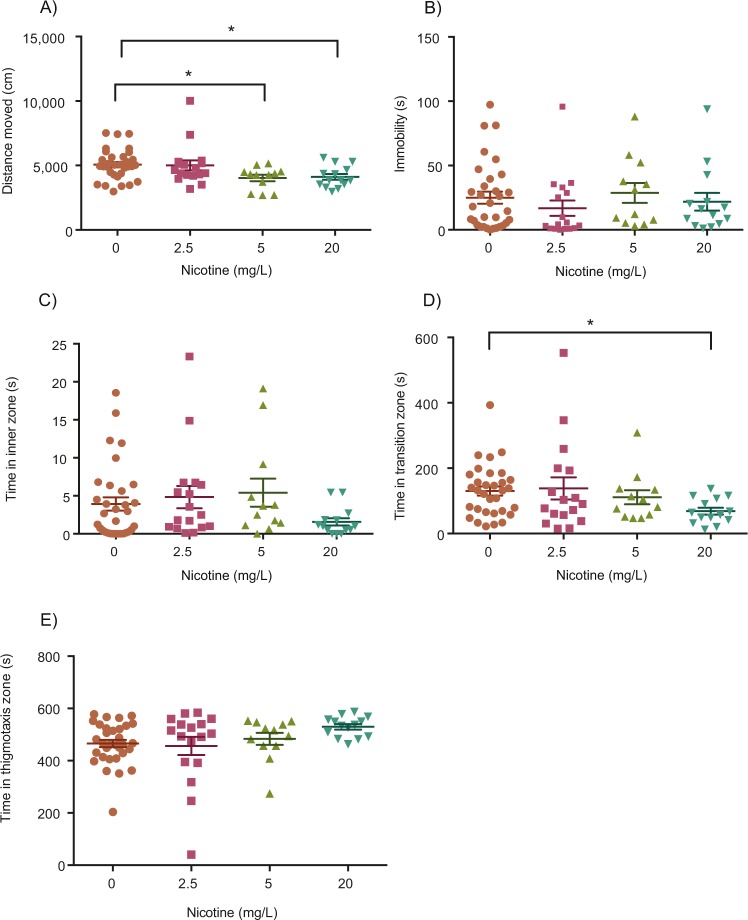
Effect of repeated nicotine exposure on locomotion and anxiety assessed after two days of withdrawal. Repeated exposure to 5 and 20 mg/L of nicotine significantly decreased total distance moved during withdrawal (**A**) but had no effect on immobility (**B**). Time in inner zone (**C**) was also not significantly affected by any dose, however, 20 mg/L of RNE significantly decreased time spent in the transition zone (**D**) and increased time spent in the thigmotaxis zone (**E**) during withdrawal. *P < 0.05.

In a third experiment, zebrafish were repeatedly exposed to 0, 2.5, 5, or 20 mg/L of nicotine then tested two days after their last drug exposure. A significant difference was found between the control, 5 and 20 mg/L groups in total distance moved (Fig. 4A; Kruskal-Wallis; H(3) = 10.95, *p* = 0.0120, η^2^ = 0.112). Post-hoc testing indicated a significant reduction in total distance moved with the 5 mg/L (Fig. 4A; Dunn’s; *p* = 0.0238, n = 12) and 20 mg/L (Fig. 4A; Dunn’s*; p* = 0.0306, n = 14) dose of nicotine. Repeated exposure to 2.5, 5, and 20 mg/L nicotine did not have a significant effect on immobility (Fig. 4B; Kruskal-Wallis; H(3) = 4.244, *p* = 0.2363, η^2^ = 0.018) nor on the time spent in the inner (Fig. 4C; Kruskal-Wallis; H(3) = 4.892, *p* = 0.1799, η^2^ = 0.027) or thigmotaxis zones (Fig. 4E; Kruskal-Wallis; H(3) = 7.343, *p* = 0.0617, η^2^ = 0.061), however, there was a significant difference in time spent in the transition zone (Fig. 4D; Kruskal-Wallis; H(3) = 7.335, *p* = 0.0620, η^2^ = 0.061). Post-hoc testing identified a significant decrease in time spent in the transition zone (Fig. 4D; Dunn’s; *p* = 0.0204, n = 14) for the 20 mg/L group.

### Repeated nicotine exposure – Experiment 2

In a fourth experiment we repeated the 21-day RNE exposure with 0 and 20 mg/L conditions and began testing fish one hour after the last dose. Compared to controls, repeated exposure to 20 mg/L of nicotine did not have a significant effect on distance moved (Mann Whitney; *U* = 350, *p* = 0.8326, η^2^ = 0.001) or immobility (Mann Whitney; *U* = 283, *p* = 0.1705, η^2^ = 0.035). The time fish spent in each of the zones (inner, transition and thigmotaxis) was however, significantly affected by RNE. Repeated exposure to 20 mg/L of nicotine significantly reduced the amount of time spent in the inner (Mann Whitney; *U* = 212.5, *p* = 0.0082, η^2^ = 0.035) and transition zones (Mann Whitney; *U* = 229, *p* = 0.0201, η^2^ = 0.099) and increased the amount of time spent in the thigmotaxis zone (Mann Whitney; *U* = 229.0, *p* = 0.0082, η^2^ = 0.099).

## Discussion

Nicotine is an addictive substance that has a complex impact on emotional state that varies with dose, duration of exposure, species tested, and within-population individual differences. Acute exposure to nicotine consistently reduces anxiety in most animals, whereas an increase in anxiety is commonly observed following withdrawal from nicotine. In this study, we focused on boldness and observed a dose-dependent increase in zebrafish following either short (three minute) or long (60 minute) acute exposures. On the contrary, a long schedule of repeated nicotine exposure caused a decrease in boldness and movement during withdrawal.

In this study, we used the novel object approach test to examine boldness after acute nicotine exposure and RNE. This test involves exposing fish to a novel object positioned in the center of an arena and the quantification of time spent near the object as a proxy for boldness. Boldness and anxiety-like behaviour are very intertwined; however, they may be regulated by different neurotransmitter systems. Pharmacological compounds that normally alter anxiety, such as caffeine^33^ and the GABA_A_-receptor antagonist, gabazine^34^ have been studied with the novel object approach test and both increased the time fish spent in the thigmotaxis zone. Conversely, anxiolytic drugs like ethanol increase the time spent in the transition zone near the novel object^35,36^. Therefore, increased object exploration (more time spent in transition or inner zones and less time in the thigmotaxis zone) are likely to be indicative of reduced anxiety in zebrafish. However, to validate changes in anxiety-like behaviour additional tests like the light/dark test or novel tank dive test should be performed, and therefore, the results from our study will only be used to discuss changes in boldness via the novel object approach test. We found that acute exposure to nicotine at 25, 50, 100 or 400 mg/L for three minutes had no significant effect on the amount of time fish spent in the inner zone. However, fish exposed to 100 or 400 mg/L of nicotine for three minutes spent significantly more time in the transition zone and less time in the thigmotaxis zone. When exposed to lower doses of nicotine for a longer period of time (60-minutes), 20 mg/L of nicotine significantly increased the amount of time fish spent in both the transition and inner zones and decreased time in the thigmotaxis zone. This increased proximity to the novel object is consistent with findings from previous studies where lower doses (4 and 8 mg/L) reduced shoal cohesion, a measure of increased exploration in adult zebrafish^24^. Thus, our data confirms a dose-dependent increase in boldness from acute nicotine exposure with short (3-minute) high doses (100, 400 mg/L) or long (60-minute) low dose (20 mg/L) exposures in zebrafish. However, short and long exposures did vary in some parameters that should considered. There was a significant increase in time in the inner zone for long-low dose exposures (Fig. 3C) but not short-high dose exposures (Fig. 2C) which suggests that a long-low dose exposure is more effective at increasing boldness. Furthermore, a short exposure of 50 mg/L increased immobility (Fig. 2B) whereas there were no effects of long exposures on locomotion (Fig. 3B). Notably, we administered the novel object approach test immediately after dosing, consistent with other research using this test^34–37^, however, the peak effects of nicotine with 3-minutes of exposure may be not be observed until 20–40 minutes later^18^.

In humans, nicotine withdrawal can have a profound impact on well-being as common symptoms include anxiety, depressed mood, irritability, insomnia, difficulty concentrating, and restlessness^38^. Taken together, these emotional symptoms are termed the ‘affective withdrawal symptoms’^39^. To date, there have been a number of potential treatments including nicotine replacement therapies in the form of lozenges or patches^39^, as well as the recent development of nicotine vaccines to help alleviate cravings^40^. To test whether these treatments are efficacious, the rodent model is most commonly used. Rodents undergo chronic or repeated nicotine exposures that, upon cessation, lead to characteristic withdrawal symptoms that can be subdivided into somatic or affective/cognitive symptoms^41,42^. Somatic withdrawal symptoms include changes in physical movements such as chewing, teeth-chattering, shaking, tremors, whrithing, palpebral ptosis, gasping and yawning (reviewed in^42^). Affective/cognitive withdrawal symptoms are more complex and require various behavioural tests for anhedonia, anxiety, and irritability. Common tests for anxiety are the elevated plus maze and light/dark test that have both been used to demonstrate an increase in anxiety during nicotine withdrawal in rodents^10,43,44^, however, other studies have found no change in anxiety during withdrawal^45^. RNE and addiction have been studied in zebrafish using conditioned place preference tests^26,27^, however, none have examined withdrawal behaviours and boldness.

**Figure 5:**
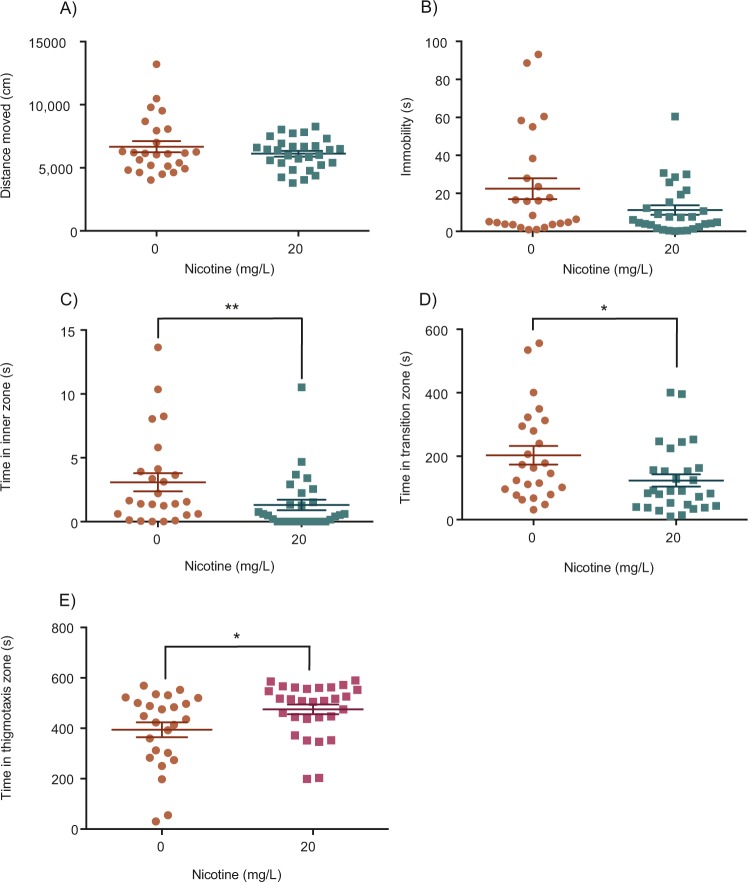
Effect of repeated nicotine exposure on locomotion and anxiety assessed one hour after the final exposure. Repeated exposure to 20 mg/L of nicotine did not have a significant effect on distance moved (**A**) or immobility (**B**). The time spent in the inner (**C**) and transition (**D**) zones were significantly reduced by repeated exposure to 20 mg/L of nicotine, whereas repeated exposure to 20 mg/L of nicotine increased the time spent in the thigmotaxis zone (**E**). ***P < 0.001; **P < 0.01; *P < 0.05.

In our study, we observed RNE-induced changes in distance moved that were only observed following two days of withdrawal. Withdrawal-induced changes in locomotion are not uncommon in zebrafish after exposure to other substances like cocaine, morphine, and alcohol^46^. In our study, fish repeatedly exposed to either 5 or 20 mg/L of nicotine moved less throughout trials when tested two-days after the cessation of nicotine exposure (Fig. 4), whereas there was no effect of altered locomotion when tested on day 21 of RNE (Fig. 5). These finding parallel depressant effects observed in rodents following a 16-hour period of nicotine abstinence^47^ and suggests that nicotine withdrawal caused changes in locomotion in our study.

With acute administration, we observed no changes in distance moved in zebrafish exposed to nicotine for three minutes or for 60 minutes. This is consistent with previous research from our lab with 50 mg/L^21^, however, other studies employing a novel tank diving paradigm have found similar doses increase^48^ as well as decrease^23^ total distance moved. It is unclear why there is a discrepancy in locomotion due to acute nicotine exposure, however, this may be due to the behavioural test, or other differences in experimental conditions and dosing procedures. Nonetheless, in the current study we observed only a decrease in distance moved during withdrawal from RNE.

In terms of boldness and withdrawal, we observed a decrease in boldness (less time in the center zone and more time in the thigmotaxis zone) immediately following RNE, which suggests an aversive response due to nicotine. Two days after the last RNE exposure there was still decreased time in the transition zone and a similar, but not significant, effect on time in the inner and transition zones. Future research should examine the duration of the RNE-induced withdrawal state in zebrafish and determine when this state is most severe with multiple behavioural and hormonal (eg. cortisol) measures. The next logical step is to use putative pharmacological manipulations to relieve the decreased boldness (and possibly increased anxiety) to help develop clinically relevant treatments for nicotine addiction such as nicotine replacement therapy, buproprion, varenicline, and nicotine vaccines^42^.

## Conclusion

This study provides the first evidence of an RNE-induced alteration of boldness and locomotion in zebrafish. Zebrafish are a powerful tool that can be used to examine pharmacologically induced changes in brain circuitry after repeated drug exposure. Future studies should investigate neuroadaptation that occurs with RNE and whether other agents can be used to reverse these behavioural symptoms.

## Materials and methods

### Animals and housing

Wild-type (short-fin) zebrafish (*Danio rerio*) were acquired from Aquatic Imports (Calgary, AB) and were a minimum age of 9-months and a mixture of males and females (~50:50 ratio, n = 305). All fish were experimentally naïve and housed at a maximum density of 15 fish per 3 L polypropylene tank. Tanks were stored in a three-shelf bench top system which was controlled for filtration and aeration (Aquatic Habitats, Aquatic Ecosystems, Inc. Apopka, FL, USA). Habitat water was buffered with non-iodized salt, sodium bicarbonate, and acetic acid and was maintained at a pH between 6.8 and 7.5. Temperature remained consistent between 26.0 and 30.0 °C and ten percent water changes were performed daily. Husbandry was as described previously^29^. Briefly, all fish were maintained on a 12-hr light/dark cycle, with lights on at 8AM and off at 8PM. A cal SPOT 401 photometer (Cooke Corp. CA, USA) was used to measure luminance in the habitat tanks when the lights were on (33 cd/m^3^). Fish were fed fish pellets (Gemma Micro 300, Skretting by Nutreco, France) and dry brine shrimp (Omega One Freeze Dried Mysis Shrimp nutri-treat, OmegaSea Ltd., Germany) once per day (after dosing during experiments). All experiments were approved by the MacEwan University Animal Research Ethics Board (AREB) under protocol number 05-12-13, in compliance with the Canadian Council for Animal Care (CCAC) guidelines for the care and use of experimental animals. All animals were drawn from the same batch of fish in each of the experiments.

### Nicotine administration

#### Three-minute acute nicotine administration

Fish were randomly assigned to either a control group (n = 18), or to one of four experimental groups, with sample sizes based on previous studies^16,18,20^. Experimental groups were exposed to either 25 (n = 15), 50 (n = 15), 100 (n = 15), or 400 mg/L (n = 15) of (-) nicotine hydrogen tartrate salt (Sigma-Aldrich, Oakville, ON, Canada) for three minutes. Doses were based on similar behavioural experiments where nicotine had a significant behavioural effect^21,23,49^ and solutions were made fresh each day with habitat water. Prior to feeding, fish were individually netted from their housing tanks and placed into a 500 mL dosing beaker containing the respective nicotine concentrations (ie. 25, 50, 100, or 400 mg/L) in 400 mL of solution for the three-minute duration. White corrugated plastic was set up around the dosing beaker and habitat tanks to limit external stimuli. A small square piece of the plastic was placed on top of the dosing beaker to ensure fish remained inside and to prevent evaporation of the nicotine solution. Water temperatures were maintained between 26 and 30 °C by seedling heat mats (Hydrofarm Horticultural Products, Petaluma CA) positioned beneath the dosing beaker and habitat tanks. Fish were immediately netted after the three minutes and placed into the arena for behavioural testing. Control fish were exposed to the same protocol with the exception of the absence of nicotine in the dosing beaker.

#### 60-minute acute nicotine administration

Fish were randomly assigned to either a control group (n = 16) or to one of four experimental groups exposed to either 2.5 (n = 15), 5 (n = 15), or 20 (n = 15) mg/L of nicotine for 60 minutes. These doses are 20 times less than the 3-minute experiment (50, 100, 400 mg/L, respectively), but administered for a 20 times longer duration. Prior to feeding, fish were individually netted from the habitat tanks and placed into one of four 3 L dosing tanks containing spawning inserts^16^, 1.5 L of habitat water, and either 0, 2.5, 5, or 20 mg/L of nicotine. The dosing tanks were covered by clear plastic lids. Dosing and habitat tanks were surrounded by a white corrugated plastic barrier and water temperatures were kept constant. At the end of the 60 minutes, fish were transferred using the spawning inserts into the arena for behavioural testing.

### Repeated nicotine administration - Experiment 1

Fish were randomly assigned to a control group (n = 39) or to one of four experimental groups exposed to either 2.5 (n = 20), 5 (n = 18), or 20 mg/L (n = 20) of nicotine for 60 minutes. Random allocation was performed using a random number generator in Microsoft Excel. Each treatment consisted of two replicate groups and the controls contained four, with sample sizes ranging from 4-10 for each replicate group. All fish were held in spawning inserts in the habitat within 3 L tanks^16^. Dosing tanks^16^ were positioned on a flat surface before nicotine was added to 1.5 L of habitat water. Next, housing tanks were placed in-front of the corresponding dosing tanks and after a ten-minute habituation period, each group of fish was transferred into the dosing tanks using the spawning inserts. Dosing began at approximately 12:00 PM each day (±30 minutes). Fish remained in the covered dosing tanks for 60 minutes and were surrounded by a white corrugated plastic barrier to avoid extraneous visual stimulation. Water temperatures were maintained between 26 and 30 °C. Following the 60-minute dosing period, fish were transferred with the spawning inserts back into the housing tanks^16^. Control fish were exposed to the same protocol with the exception that the dosing tanks only contained habitat water. Fish were fed approximately 30 minutes after dosing. This dosing procedure (Fig. 1A) was repeated prior to feeding each day for 21 consecutive days and behavioural testing (Fig. 1B–D) occurred two days after the final dose was administered.

### Repeated nicotine administration - Experiment 2

To determine whether nicotine has a lasting effect that is still present after two days of withdrawal, or whether zebrafish experience nicotine withdrawal that develops two days later, we performed an additional experiment where we repeatedly exposed experimentally naive fish to either 0 (n = 29) or 20 mg/L (n = 30) of nicotine, identical to the previous experiment. However, unlike the previous RNE experiment where fish were tested after a two-day withdrawal period, fish in this experiment were tested one hour after the last nicotine exposure (Fig. 1B). Two replicate groups were used for each condition with sample sizes ranging from 12 to 15 per replicate group.

### Experimental apparatus and behavioural testing

The novel object approach test (Fig. 1C–E) was used to test anxiety-like behaviour in the fish. This test uses the avoidance or exploration of a novel object in the center of an arena to quantify boldness/anxiety. Avoidance of the novel object is indicative of decreased boldness  whereas, increased object exploration is suggestive of increased boldness^34,35,50^. All experimental procedures were conducted between 8AM and 6PM in a room with diffuse overhead lighting. To limit external stimuli during testing, a three-sided enclosure constructed out of white corrugated plastic was positioned around the arena and the experimental room was controlled for sound. The testing arena (Fig. 1C) used in this study was an opaque, white coloured circular arena with a diameter of 34 cm and depth of 15 cm. A Lego figurine (2 cm × 4.25 cm; Fig. 1D) which was multi-coloured and identical to previous studies^34–37^ was used to rule out any possible colour preferences^51^. This novel object was positioned in the center of the arena. The arena was then filled with 6 cm of habitat water which was changed at least every four hours and maintained at a temperature between 26 and 30 °C using seedling heat mats under the arena. Additionally, temperature was measured at the start of each trial with water exchanged for an equal amount of heated habitat water if the temperature fell below 26 °C. Ethovision XT motion tracking software (version 7.0, Noldus, VA, USA) was used to record the distance traveled and time spent in each virtual zone (Fig. 1E) representing the thigmotaxis, transition and inner zones for each 10-minute trial. Immobility was quantified with a 5% threshold in Ethovision, meaning any less than a 5% change in the pixels of the detected fish between frames would count as immobile^52^. Fish exposed to acute levels of nicotine were placed immediately into the testing arena following the three or 60-minute exposure periods. For RNE experiments, fish that were tested two days after the final exposure were transferred in the habitat tanks onto seedling heat mats positioned outside the barrier and were given 10 minutes to habituate to this environment before being placed into the arena. Fish that were tested one hour after the final dose followed the same protocol but remained in their habitat tanks for one hour prior to testing. Recording began immediately after individual fish were placed into the transition zone, facing the object. Following each 10-minute trial, fish were netted and placed into a holding tank.

### Statistical analysis

Data was analyzed using GraphPad Prism software (CA, USA). Normality was assessed using D’Agostino & Pearson omnibus normality tests. For the acute experiments and the first RNE experiment, when the normality test gave no evidence to suggest that the data was not normal, one-way ANOVA and Dunnett’s multiple comparison tests were used to test for significant differences between the groups of data. When the normality test gave evidence to suggest the data was not normal, the Kruskal-Wallis test and Dunn’s multiple comparison tests were used. For the second RNE experiment the Mann-Whitney U test was used to test for significant differences between the groups found for distance moved, immobility, and time spent in the inner, transition, and thigmotaxis zones. All tests were conducted at the 5% significance level (i.e, α = 0.05). When applicable, the effect size for each the considered test is given. For consistency, the effect size is reported using the η^2^ value^53–55^. The effect size can be interpreted as follows: 0–0.010 indicates no effect, 0.010–0.060 indicates a small effect, 0.060–0.140 indicates an intermediate effect, and 0.140–0.200 indicates a large effect^53,54^. No significant differences were found between replicate groups so these were combined for analysis. Fish that were immobile for 100 seconds or more were removed from the analysis (Acute 3-min: control n = 3; 25 mg/L n = 2; 50 mg/L n = 2; 100 mg/L n = 2; 400 mg/L n = 1; Acute 60-min: control n = 3; RNE experiment 1: control n = 7; 2.5 mg/L n = 3; 5.0 mg/L n = 6; 20 mg/L n = 6; RNE experiment 2: control n = 4; 20 mg/L n = 1). All data are available in the supplementary material section.
